# Letter to the editor regarding “Epidemiologic analysis of 8000 acute vertebral fractures: evolution of treatment and complications at 10-year follow-up”

**DOI:** 10.1186/s13018-022-03279-y

**Published:** 2022-08-12

**Authors:** Dietrich Doll, Markus M. Luedi

**Affiliations:** 1 Department of Surgery, St. Mareinhospital Vechta, Vechta, Germany; 2 Department of Anaesthesiology and Pain Medicine, Inselspital, Bern University Hospital, University of Bern, Bern, Switzerland

## Abstract

In this letter to the editor, we discuss the article by Bigdon et al., published recently in the *Journal of Orthopaedic Surgery and Research, *about their accurate single-centre cohort study of 8000 vertebral fractures in 4772 patients. As the complication rate of this cohort is low, it seems that severe trauma patients needing damage control resuscitation/procedures may have undergone damage control in the first treating hospital before being transferred to the trauma centre. It will be interesting to see how both activity and health trends within the ageing population will change osteoporotic occurrence of fractures and enable more conservative trends versus operative stabilization to continue an active life even in the seventh or eighth decade.

Dear Editor

We have studied the article by Bigdon et al., published recently in the *Journal of Orthopaedic Surgery and Research*, with great interest [1]. The authors analysed *n* = 330,225 emergency room patients and identified more than 8000 vertebral fractures in 4772 patients over a 10-year period. Although this is a retrospective single-centre cohort study (University Hospital serving as a Level 1 trauma centre), the methodology is accurate in choosing an observation period that excludes any study surgeon from having treated the patients and their analysed complications. The authors furthermore excluded patients below age 16 and patients with inadequate data sets. In a stringent analysis separating traumatic from osteoporotic and neoplastic fractures, the authors outline trends and complication rates. Although vertebral pathology was well defined, with further analysis based on it, the occurrence and extent of concomitant trauma sequelae was not explicitly mentioned. From other trauma studies we know that the need for damage control procedures or a higher amount of tissue trauma leading to damage control procedures may change outcome, complication rate, and mortality substantially [2–4]. As the complication rate of this cohort is low, it seems that severe trauma patients needing damage control resuscitation/procedures may have undergone initial resuscitation in the first treating hospital before being transferred to the trauma centre. Falls from a height that led to spinal injury with multiple vertebral fractures, as seen in this cohort, may also be accompanied by cerebral injury [5, 6]. In this cohort, however, this seems to be rare to non-existent.

The authors are very correct in highlighting the changes in treatment approaches, since 5 studies are questioning vertebro/kyphoplasty, despite being a surgical trend with many departments flourishing through the “cementation” of collapsed vertebral fractures. So, based on the treatment trends highlighted by Bigdon et al., it will be interesting to see how activity and health trends within the ageing population will change occurence of osteoporotic fractures and enable more conservative treetments versus operative stabilization to continue an active life even in the seventh or eighth decade.

## Data Availability

Not applicable.
